# The risk of psoriasis in patients with uveitis: A nationwide population-based cohort study

**DOI:** 10.1371/journal.pone.0255492

**Published:** 2021-08-02

**Authors:** Yu-Yen Chen, Hsin-Hua Chen, Tzu-Chen Lo, Pesus Chou

**Affiliations:** 1 School of Medicine, National Yang Ming Chiao Tung University, Taipei, Taiwan; 2 Department of Ophthalmology, Taichung Veterans General Hospital, Taichung, Taiwan; 3 School of Medicine, Chung Shan Medical University, Taichung, Taiwan; 4 Community Medicine Research Center and Institute of Public Health, National Yang Ming Chiao Tung University, Taipei, Taiwan; 5 National Chung Hsing University, Taichung, Taiwan; 6 Division of Allergy, Immunology, and Rheumatology & Division of General Internal Medicine, Department of Internal Medicine, Taichung Veterans General Hospital, Taichung, Taiwan; 7 Institute of Biomedical Science and Rong-Hsing Research Center for Translational Medicine, Chung Hsing University, Taichung, Taiwan; 8 Department of Industrial Engineering and Enterprise Information, Tunghai University, Taichung, Taiwan; 9 Department of Medical Education, Taichung Veterans General Hospital, Taichung, Taiwan; Universita Campus Bio-Medico di Roma, ITALY

## Abstract

**Objective:**

To evaluate whether the risk of subsequent psoriasis and psoriatic arthritis development is increased in patients with uveitis.

**Methods:**

In Taiwan’s national health insurance research database, we identified 195,125 patients with new-onset uveitis between 2001 and 2013. We randomly selected 390,250 individuals without uveitis who were matched 2:1 to uveitis cases based on age, sex and year of enrolment. The characteristics of the two groups were compared. Using multivariate Cox regression, hazard ratios (HRs) for psoriasis or psoriatic arthritis corresponding to uveitis were computed after adjustment for age, sex, insurance cost and comorbidities. In subgroup analyses, separate HRs for mild psoriasis, severe psoriasis and psoriatic arthritis were calculated.

**Results:**

The mean age of the study cohort was 50.2 ± 17.2 years. Hypertension, diabetes, hyperlipidaemia and obesity were more prevalent in the uveitis group (all *p* < 0.0001). The hazard of psoriasis or psoriatic arthritis development was significantly greater in the uveitis group than in the non-uveitis group (*p* < 0.0001); this increased risk persisted after adjustment for confounders [adjusted HR = 1.41; 95% confidence interval (CI), 1.33–1.48]. Adjusted HRs showed an increasing trend from mild psoriasis (1.35; 95% CI, 1.28–1.44) to severe psoriasis (1.59; 95% CI, 1.30–1.94) and psoriatic arthritis (1.97; 95% CI, 1.60–2.42).

**Conclusions:**

This nationwide population-based cohort study revealed that patients with uveitis have an increased risk of subsequent psoriasis or psoriatic arthritis development.

## Introduction

Uveitis is an intraocular inflammatory disease with many causes, such as infection, immune reaction, genetic predisposition and environmental precipitation. It is usually classified according to the main location of involvement in the eye. In anterior uveitis, leukocytes in the anterior chamber of the eye may be visible on slit-lamp examination. The intermediate and posterior forms of uveitis are diagnosed upon direct visualisation of chorioretinal inflammation and/or leukocytes in the vitreous by ophthalmoscopy. Higher levels of cytokines, such as interleukin (IL)-17 and tumour necrosis factor (TNF)-α, have been found in the aqueous humour of patients with uveitis, suggesting that T [T helper (Th)1 and Th17] cells are involved in the immunopathogenesis of the disease [1, 2]. In addition, genetic studies have revealed a link between human leukocyte antigen (HLA)-B27 and uveitis [3, 4], indicating that uveitis may be a manifestation of systemic diseases associated with this antigen, such as spondyloarthropathies and reactive arthritis [5–8].

Psoriasis is a chronic, systemic, inflammatory autoimmune disease characterised by thick scaling skin plaques. A reported 6–42% of patients with psoriasis have psoriatic arthritis [9, 10], a spondyloarthropathy. Psoriasis is promoted by Th1 and Th17 cells, with cytokines such as IL-6, IL-12, IL-17, IL-22, IL-23 and TNF-α playing substantial roles in the inflammatory response [11]. In addition, large cohort studies have revealed associations between psoriasis and hypertension [12], diabetes [12, 13], hyperlipidaemia [12, 14] and obesity [12]. Some occurrence of these comorbidities correlate positively with psoriasis severity [13, 15]. All of these findings imply that psoriasis should be regarded as a disease related to various organs, and not limited to the skin and joints.

The pathogeneses of uveitis and psoriasis are similar, involving inflammation and immune reactions. Several studies have been conducted to investigate the risk of uveitis in patients with psoriasis. Chandran *et al*. [16] reported that 2 (2%) of 100 patients with psoriasis in Singapore had uveitis, and that the occurrence of uveitis was associated independently with the severity of psoriasis in this sample. Hospital-based cross-sectional studies conducted in Turkey, Iran and Egypt have revealed nonsignificant trends toward a higher prevalence of psoriasis among patients with than among individuals without uveitis [17–19]. In contrast, large-scale population-based studies, including those with cross-sectional and cohort designs [20–23], have demonstrated that the risk of uveitis is significantly greater among patients with than among individuals without psoriasis. The inconsistency in findings may be attributable to the examination of small numbers of cases in the hospital-based studies.

In this study, we used a nationwide population-based database to investigate the association between uveitis and psoriasis in patients in Taiwan. Using a cohort study design, we focused on patients with uveitis and determined the risk of subsequent psoriasis or psoriatic arthritis development. Very few studies have been conducted with a cohort, directional approach from uveitis to psoriasis, and most of studies to date have been performed in western countries [21, 22]. This study is one of the first to determine the risk of psoriasis or psoriatic arthritis development in an Asian uveitis population.

## Materials and methods

### Data source

Taiwan’s national health insurance (NHI) programme currently covers health care services for >99% of Taiwan’s 23 million residents. The national health insurance research database (NHIRD), maintained by the National Health Research Institutes of Taiwan and released for research purposes, contains registration and demographic data for all NHI enrolees, and health care, medical prescription, and surgical management data for patients receiving ambulatory and in-hospital care in Taiwan. For this retrospective cohort study, we used NHIRD data on health care claims for the entire population in 1996–2013. As patient identification and all information are fully encrypted before NHIRD data are released, the requirement for written informed consent was waived for this study. The ethics committee of Yang-Ming University Hospital approved the study (no. 2015A018).

### Sample, diagnostic criteria and study period

Patients newly diagnosed with uveitis were enrolled, Patients with infectious uveitis were excluded. Therefore, patients included in our study had non-infectious uveitis. Anterior, intermediate, posterior, and panuveitis were all selected, according to the International Classification of Diseases, 9th Revision, Clinical Modification (ICD-9-CM; codes 363.0x, 363.1x, 363.2x, 364.1x, 364.2x, 364.3x, 360.11, 360.12, 360.14, 362.18, 364.00, 364.01, 364.04 and 364.05), between 2001 and 2013 were selected for the uveitis group. Patients diagnosed with uveitis between 1 January 1996 and 31 December 2000 were excluded to eliminate those with existing uveitis. The index date was defined as the date of the first uveitis claim. We also randomly selected individuals who had never received a diagnosis of uveitis as a control group, at a ratio of 1:2 and matched to the uveitis group according to age, sex and index year (the year of the index date or enrolment). Patients diagnosed with psoriasis or psoriatic arthritis prior to the index year were excluded. The uveitis and control groups were followed until the end of 2013 to determine whether they subsequently developed psoriasis (ICD-9-CM codes 696.1 and 696.8) and/or psoriatic arthritis (ICD-9-CM code 696.0), diagnosed by dermatologists and rheumatologists using well-established criteria and protocols.

### Study variables

The independent variable of interest was uveitis. Demographic characteristics, such as age and sex, were extracted from the NHIRD registration files. We used the cost of insurance to represent economic status, as it is correlated with income in the NHI programme. Data on comorbidities were derived from diagnostic codes recorded in the database. The included comorbidities, adjusted due to their associations with psoriasis and psoriatic arthritis, were diabetes (ICD-9-CM code 250.x), hypertension (ICD-9-CM codes 401.x–405.x), hyperlipidaemia (ICD-9-CM code 272.x) and obesity (ICD-9-CM code 278.x).

Uveitis was classified into four types according to the location of inflammation (anterior, intermediated, posterior, and panuveitis). The outcome of interest was psoriasis or psoriatic arthritis. Psoriasis cases were classified as mild and severe according to the treatment pattern; patients with severe psoriasis should have received systemic therapy, including acitretin, methotrexate, cyclosporine, etanercept, adalimumab and ustekinumab, and/or phototherapy.

### Statistical analysis

Differences between the uveitis and control groups in age, sex, insurance cost, diabetes, hypertension, hyperlipidaemia and obesity were analysed using the two-sample *t* test (for continuous variables) and the chi-squared test (for categorical variables). A Cox proportional-hazard model was used to estimate the hazard ratio (HR) for the occurrence of psoriasis or psoriatic arthritis according to uveitis and each variable listed above in univariate and multivariate analyses. Subsequently, Cox regression analyses were applied to different forms of uveitis (anterior, intermediate, posterior, panuveitis) separately to derive the risk of psoriasis or psoriatic arthritis corresponding to each type of uveitis. Moreover, we divided the outcome variable into mild psoriasis, severe psoriasis, and psoriatic arthritis. In subgroup analyses, Cox regression was used to estimate adjusted hazard ratios (aHRs) for the occurrence of mild psoriasis, severe psoriasis and psoriatic arthritis. A *p*-value <0.05 was considered to be statistically significant. All statistical operations were performed using the SAS statistical package (version 9.4; SAS Institute, Cary, NC, USA).

## Results

### Characteristics of the study sample

In total, 195,125 patients with uveitis and 390,250 matched controls were enrolled in the study. Table 1 displays the characteristics of the two groups. The mean age was 50.2 years in both groups, and about 54% of individuals were male; the mean follow-up periods were similar (uveitis, 6.02 years; control, 6.04 years). Economic status (insurance cost) was lower in the uveitis group (*p* < 0.001). Hypertension, diabetes, hyperlipidaemia and obesity were significantly more prevalent in the uveitis group (all *p* < 0.0001). During the study period, the cumulative incidence of psoriasis and psoriatic arthritis was significantly higher in the uveitis group (1.1%) than in the control group (0.8%; *p <* 0.0001).

**Table 1:** Characteristics of the study subjects.

Variable	Total	With uveitis	Without uveitis	*p*
	(*n* = 585,375)	(*n* = 195,125)	(*n* = 390,250)	
Age, years	50.2 ± 17.2	50.2 ± 17.2	50.2 ± 17.2	1.00
Age group, years				1.00
<40	116,787 (30.2)	58,923 (30.2)	117,864 (30.2)	
40–60	221,501 (37.8)	73,830 (37.8)	147,671 (37.8)	
≥60	187,087 (32.0)	62,372 (32.0)	124,715 (32.0)	
Sex				1.00
Male	313,815 (53.6)	104,602 (53.6)	209,213 (53.6)	
Female	271,560 (46.4)	90,523 (46.4)	181,037 (46.4)	
Insurance cost (NTD)				0.0003
<20000	330,410 (56.4)	110,742 (56.8)	219,668 (56.3)	
20000–40000	170,088 (29.1)	56,054 (28.7)	114,034 (29.2)	
≥40000	84,877 (14.5)	28,329 (14.5)	56,548 (14.5)	
Hypertension				<0.0001
Yes	226,404 (38.7)	82,439 (42.3)	143,965 (36.9)	
No	358,971 (61.3)	112,686 (57.7)	246,285 (63.1)	
Diabetes				<0.0001
Yes	120,211 (20.5)	49,735 (25.5)	70,476 (18.1)	
No	465,164 (79.5)	145,390 (74.5)	319,774 (81.9)	
Hyperlipidaemia				<0.0001
Yes	165,585 (28.3)	63,356 (32.5)	102,229 (26.2)	
No	419,790 (71.7)	131,769 (67.5)	288,021 (73.8)	
Obesity				<0.0001
Yes	12,865 (2.2)	5037 (2.6)	7828 (2.0)	
No	572,510 (97.8)	190,088 (97.4)	382,422 (98.0)	
FU duration, years	6.03 ± 3.7	6.02 ± 3.7	6.04 ± 3.7	0.23
PS/PSA during FU	5340 (0.91)	2157 (1.1)	3183 (0.8)	<0.0001
Time to PS/PSA	4.21 ± 2.99	4.20 ± 2.97	4.21 ± 3.00	0.15

Data are presented as means ± standard deviations or *n* (%). NTD, new Taiwan dollars; FU, follow-up; PS, psoriasis; PSA = psoriatic arthritis. Time to PS/PSA means the timeframe between the first uveitis episode and the onset of PS/PSA.

### Univariate and multivariate Cox regression results

The unadjusted HR for psoriasis or psoriatic arthritis was 1.40 times greater in the uveitis group than in the control group [95% confidence interval (CI), 1.32–1.48; *p* < 0.0001; Table 2]. After adjustment for covariates, this significantly greater hazard remained (aHR = 1.41; 95% CI, 1.33–1.48; *p* < 0.0001). Age was a significant risk factor for psoriasis or psoriatic arthritis in univariate and multivariate analyses; the aHR for individuals aged ≥ 60 years was 1.96 (95% CI, 1.81–2.13) relative to those aged < 40 years (*p* < 0.0001). Men were more likely than women to develop psoriasis or psoriatic arthritis (aHR = 1.36; 95% CI, 1.29–1.44; *p* < 0.0001). The risk of developing psoriasis or psoriatic arthritis was significantly lesser among patients with higher insurance costs (*p* < 0.05). This risk was significantly greater among individuals with hypertension (aHR = 1.12; 95% CI, 1.06–1.19; *p* = 0.02), but not among those with diabetes, hyperlipidaemia or obesity, in multivariate analyses.

**Table 2:** Risk factors for PS and PSA in patients with and without uveitis.

Predictive variable	Univariate analysis		Multivariate analysis	
	Unadjusted HR (95% CI)	*P*	Adjusted HR (95% CI)	*p*
Uveitis (yes vs. no)	1.40 (1.32–1.48)	<0.0001	1.41 (1.33–1.48)	<0.0001
Age, years				
<40	Reference		Reference	
40–60	1.25 (1.17–1.35)	<0.0001	1.35 (1.25–1.45)	<0.0001
≥60	1.81 (1.69–1.95)	<0.0001	1.96 (1.81–2.13)	<0.0001
Sex (male vs. female)	1.29 (1.22–1.36)	<0.0001	1.36 (1.29–1.44)	<0.0001
Insurance cost, NTD				
<20000	Reference		Reference	
20000–40000	0.84 (0.79–0.90)	<0.0001	0.92 (0.86–0.98)	0.01
≥40000	0.79 (0.73–0.86)	<0.0001	0.85 (0.78–0.92)	0.0002
Hypertension (yes vs. no)	1.20 (1.14–1.27)	<0.0001	1.12 (1.06–1.19)	0.02
Diabetes (yes vs. no)	1.13 (1.06–1.20)	0.0002	1.03 (0.96–1.11)	0.43
Hyperlipidaemia (yes vs. no)	1.06 (0.99–1.12)	0.06	1.03 (0.98–1.10)	0.06
Obesity (yes vs. no)	1.00 (0.84–1.20)	1.00	1.12 (0.94–1.35)	0.21

PS, psoriasis; PSA, psoriatic arthritis; NTD, new Taiwan dollar. Multivariate analyses were adjusted for all other variables listed in the table.

### Risk of psoriasis or psoriatic arthritis according to different types of uveitis

Among all the enrolled uveitis patients, 17473 had anterior uveitis, 5854 had intermediate uveitis, 9071 had posterior uveitis, and 12727 had panuveitis. Table 3 shows the risk for psoriasis or psoriatic arthritis corresponding to each type of uveitis. All kinds of uveitis significantly increased the hazard for psoriasis or psoriatic arthritis, regardless of univariate or multivariate analyses. Intermediate uveitis had the highest risk (aHR = 1.84), followed by posterior uveitis (aHR = 1.58), panuveitis (aHR = 1.45), and anterior uveitis (aHR = 1.37).

**Table 3:** Risk for PS and PSA in patients with different types of uveitis.

Predictive variable	Univariate analysis		Multivariate analysis	
	Unadjusted HR (95% CI)	*P*	Adjusted HR (95% CI)	*P*
Anterior uveitis	1.37 (1.29–1.45)	<0.0001	1.37 (1.30–1.46)	<0.0001
Intermediate uveitis	1.79 (1.47–2.19)	<0.0001	1.84 (1.50–2.24)	<0.0001
Posterior uveitis	1.64 (1.37–1.98)	<0.0001	1.58 (1.32–1.91)	<0.0001
Panuveitis	1.37 (1.16–1.62)	<0.001	1.45 (1.23–1.72)	<0.0001

PS, psoriasis; PSA, psoriatic arthritis. Multivariate analyses were adjusted for age, sex, insurance cost, hypertension, diabetes, hyperlipidaemia, and obesity.

### Risks of mild psoriasis, severe psoriasis and psoriatic arthritis corresponding to uveitis

**Fig 1:**
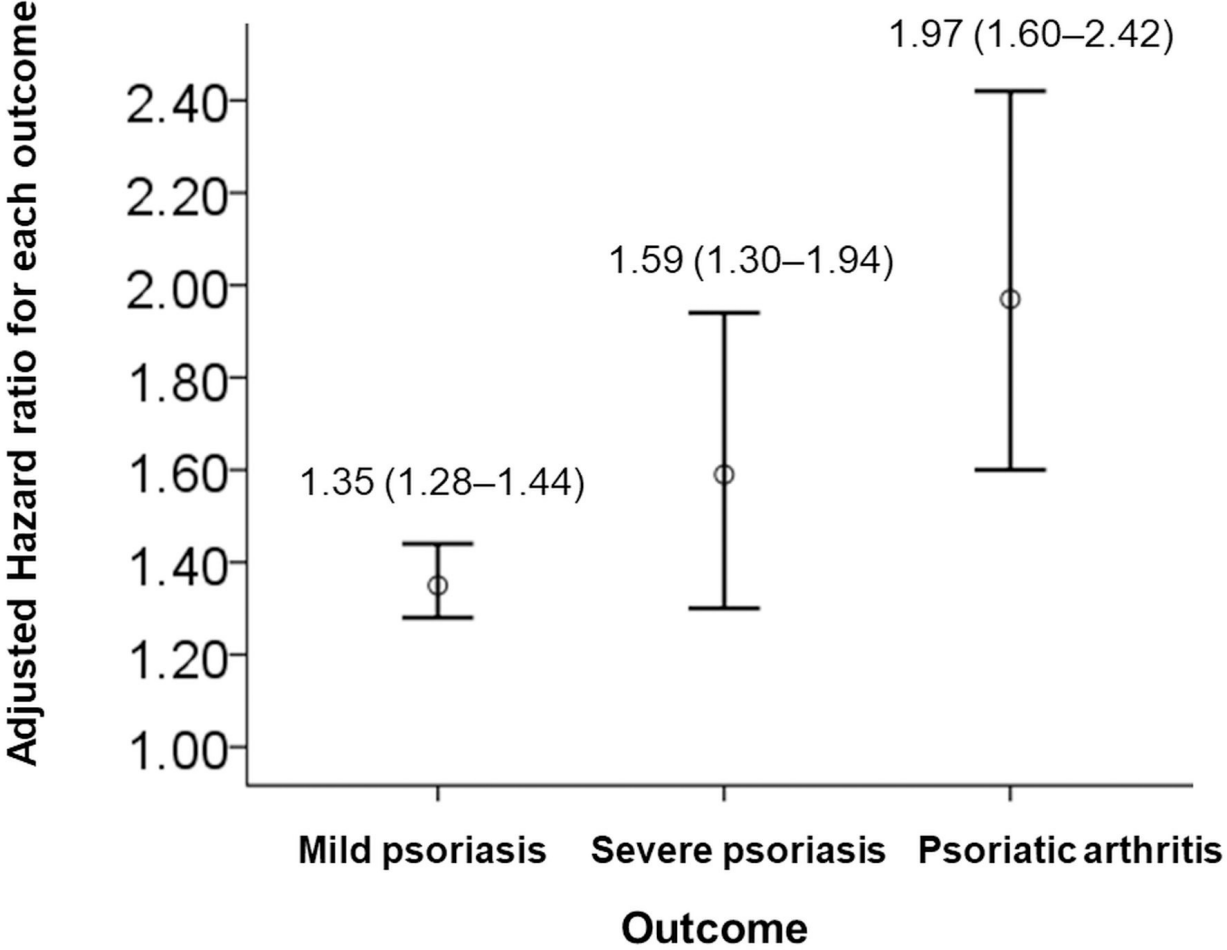
Adjusted hazard ratios for mild psoriasis, severe psoriasis and psoriatic arthritis.

Compared with the control group, the uveitis group had significantly greater risks of developing mild psoriasis (aHR = 1.35; 95% CI, 1.28–1.44), severe psoriasis (aHR = 1.59; 95% CI, 1.30–1.94) and psoriatic arthritis (aHR = 1.97; 95% CI, 1.60–2.42; Fig 1). These HRs show an increasing trend from mild to severe psoriasis and psoriatic arthritis.

## Discussion

In this 13-year follow-up study conducted with population-based data from Taiwan’s NHIRD, patients with uveitis had a significantly greater risk of developing psoriasis or psoriatic arthritis than individuals without uveitis. This risk increased with the severity of psoriasis manifestations, similar to findings from previous studies [16, 23–25].

The mean age and sex of patients with uveitis in this study are compatible with those in another population-based study conducted in Taiwan [26]. In addition, we found that hypertension, diabetes, hyperlipidaemia and obesity were significantly more prevalent in patients with uveitis than in the non-uveitis group. Previous studies also have revealed associations between uveitis and these comorbidities [26, 27]. Since hypertension, diabetes, hyperlipidaemia and obesity are also risk factors for cardiovascular diseases, uveitis patients may have a higher possibility for developing cardiovascular diseases. This hypothesis has been verified in our previous study using the Taiwan NHIRD [28].

To date, no definite theory has been offered to explain the associations of uveitis with psoriasis and psoriatic arthritis. The release of cytokines and other mediators produced in the inflamed uvea into the systemic circulation may increase the risk of psoriatic manifestations. In addition, Th1 cells, Th17 cells and certain cytokines are involved in the pathogeneses of uveitis and psoriasis/psoriatic arthritis [11, 29–31]. Moreover, the reason for the stronger association of uveitis with psoriatic arthritis than with psoriasis is not fully understood; it may be related to the greater frequency of HLA-B27 (related to uveitis) in patients with psoriatic arthritis than in those with psoriasis. Further molecular studies are warranted to elucidate the mechanisms underlying these associations.

To our knowledge, only two other population-based cohort studies have investigated the risk of subsequent psoriasis or psoriatic arthritis corresponding to the existence of uveitis. In a nationwide cohort study performed using a Danish database, Egeberg *et al*. [21] observed significantly higher incidence rate ratios for mild psoriasis (1.59), severe psoriasis (2.17) and psoriatic arthritis (3.77) among 13,114 patients with uveitis than among 5,495,164 individuals without uveitis during the 5-year study period. Using a US commercial health insurance database, Aletaha *et al*. [22] determined that HRs for the development of psoriasis (1.6) and psoriatic arthritis (4.8) over a 9.75-year study period were significantly higher among 34,423 patients with uveitis than among 256,795,796 individuals without uveitis. These findings are compatible with ours, although Egeberg *et al*. [21] adjusted for confounders (e.g. age, sex, socio-economic status, comorbidities) in regression analyses instead of matching patients and controls, and Aletaha *et al*. [22] performed matching according to age, sex and socioeconomic status, without accounting for the confounding effects of comorbidities. One strength of our study is that we not only matched the uveitis and control groups based on age and sex, but also adjusted for the imbalance in comorbidities between groups in Cox regression analyses. Thus, our results are more reliable.

Another strength of our study is the use of the comprehensive NHIRD, in which demographic data and all diagnoses and prescriptions are recorded accurately. The National Health Administration routinely checks medical charts to ensure that patients have correct diagnoses and to confirm compatibility with claims data. Thus, the diagnoses in our data were validated. The accurate diagnoses in the database enabled us to investigate the relationship between subtypes of uveitis and psoriasis/psoriatic arthritis. In addition, the large number of cases examined provided sufficient statistical power, and the cohort study design enabled clear time-sequence association. Moreover, we examined associations with mild and severe psoriasis and psoriatic arthritis separately, whereas previous studies have focused more on the association of uveitis with psoriatic arthritis [7, 32, 33] than on that with psoriasis without psoriatic arthritis [34]. Thus, we gained more detailed insight on the impacts of uveitis on psoriasis of different severities.

A limitation of our NHIRD is the lack of information regarding halotypes. Taiwan biobank collects genetic and laboratory data and may combine with NHIRD to elucidate the specific halotypes/genetic factors which affect the relationship between uveitis and psoriasis. Another limitation of our study is that we cannot differentiate the subtypes of psoriasis (e.g., erythrodermic, pustular or acral forms) because the ICD-9-CM codes applied in our NHIRD during the study period does not classify them separately. After 2016, our NHIRD adopted ICD-10-CM codes which can define each subtype of psoriasis, Still another limitation of this study is that we did not have data of Psoriasis Area Severity Index (PASI) in our analyses. PASI is commonly used to describe the severity of psoriasis. For example, in Conforti’s study [35], psoriasis patients would be at a higher risk of cardiovascular comorbidity if they have higher PASI, or in other words, more severe psoriasis. Since our NHIRD does not contain data on psoriasis area, PASI cannot be derived from our database. However, we used treatment modalities to classify the severity of psoriasis. This alternative method has been commonly used in previous studies [23, 36–40]. Further studies with information of ICD-9-CM codes and PASI are warranted to confirm he association between uveitis and different subtypes/severities of psoriasis.

## Conclusions

Patients with uveitis are at significantly greater risk of subsequent psoriasis and psoriatic arthritis development than are individuals without uveitis. Thus, ophthalmologists should be alert to the development of psoriatic lesions when managing patients with uveitis. Rheumatologists and dermatologists should also be alert for symptoms of uveitis, which may be early indicators of psoriatic arthritis [41]. The use of a referral system and close cooperation among ophthalmologists, rheumatologists and dermatologists are important to manage these patients.
